# Decrease of miR-19b-3p in Brain Microvascular Endothelial Cells Attenuates Meningitic *Escherichia coli*-Induced Neuroinflammation via TNFAIP3-Mediated NF-κB Inhibition

**DOI:** 10.3390/pathogens8040268

**Published:** 2019-11-27

**Authors:** Nouman Amjad, Ruicheng Yang, Liang Li, Jiyang Fu, Bo Yang, Bojie Xu, Chen Tan, Huanchun Chen, Xiangru Wang

**Affiliations:** 1 State Key Laboratory of Agricultural Microbiology, College of Veterinary Medicine, Huazhong Agricultural University, Wuhan 430070, China; nouman_amjad@webmail.hzau.edu.cn (N.A.); yangruicheng@mail.hzau.edu.cn (R.Y.); 15387104546@163.com (L.L.); fujiyang921021@hotmail.com (J.F.); 13100637110@163.com (B.Y.); wulixubojie@hotmail.com (B.X.); tanchen@mail.hzau.edu.cn (C.T.); chenhch@mail.hzau.edu.cn (H.C.); 2 Key Laboratory of Preventive Veterinary Medicine in Hubei Province, The Cooperative Innovation Center for Sustainable Pig Production, Wuhan 430070, China; 3 Key Laboratory of Development of Veterinary Diagnostic Products, Ministry of Agriculture of the People’s Republic of China, Wuhan 430070, China

**Keywords:** meningitic *Escherichia coli*, brain microvascular endothelial cells, miR-19b-3p, TNFAIP3, NF-κB, neuroinflammation

## Abstract

Meningitic *Escherichia coli* can traverse the host’s blood–brain barrier (BBB) and induce severe neuroinflammatory damage to the central nervous system (CNS). During this process, the host needs to reasonably balance the battle between bacteria and brain microvascular endothelial cells (BMECs) to minimize inflammatory damage, but this quenching of neuroinflammatory responses at the BBB is unclear. MicroRNAs (miRNAs) are widely recognized as key negative regulators in many pathophysiological processes, including inflammatory responses. Our previous transcriptome sequencing revealed numbers of differential miRNAs in BMECs upon meningitic *E. coli* infection; we next sought to explore whether and how these miRNAs worked to modulate neuroinflammatory responses at meningitic *E. coli* entry of the BBB. Here, we demonstrated in vivo and in vitro that meningitic *E. coli* infection of BMECs significantly downregulated miR-19b-3p, which led to attenuated production of proinflammatory cytokines and chemokines via increasing the expression of TNFAIP3, a negative regulator of NF-κB signaling. Moreover, in vivo injection of miR-19b-3p mimics during meningitic *E. coli* challenge further aggravated the inflammatory damage to mice brains. These in vivo and in vitro findings indicate a novel quenching mechanism of the host by attenuating miR-19b-3p/TNFAIP3/NF-κB signaling in BMECs in response to meningitic *E. coli*, thus preventing CNS from further neuroinflammatory damage.

## 1. Introduction

Bacterial meningitis is an important life-threatening infection of the central nervous system (CNS) with significantly high mortality, as well as severe sequelae [1]. Despite the improving sanitation practices and antimicrobial therapy, its mortality rate reaches up to 30% and prevalence rate increases up to 1% annually, especially in some poverty-hit regions [2]. Bacterial meningitis causes irremediable damage to the CNS, and most survivors suffer from multiple neurological complications [3]. A number of pathogens possess the ability to invade the host’s CNS and stimulate neuroinflammatory responses, among which *Escherichia coli* has been reported as one of the most important Gram-negative bacilli causing meningitis, particularly in the neonatal stage [4]. The blood–brain barrier (BBB) is the specialized structural and functional barrier in the brain that not only maintains normal homeostasis of the brain microenvironment, but also blocks the invading microorganisms, as well as toxins, from entering the CNS. The BBB comprises brain microvascular endothelial cells (BMECs), pericytes, and astrocytes, and manipulations in any of these cell types compromise the BBB permeability [5]. Among these cell types, the BMECs, as the very first direct layer of the barrier, gain significant attention due to their determinative roles in maintaining BBB integrity, as well as CNS homeostasis [6].

In the development of many CNS disorders, such as Alzheimer’s disease, multiple sclerosis, HIV infection, or many CNS-invading pathogen infections, etc., the generation of proinflammatory cytokines and chemokines, matrix metalloproteases, and reactive oxygen species is largely responsible for CNS dysfunction, in which a surge of neuroinflammatory responses mediated by the increased proinflammatory cytokines and chemokines could be an important contributor [7,8,9,10]. Particularly in CNS infections, the excessive neuroinflammatory response does not help to eliminate the invading pathogens successfully, but further aggravates the infection-induced inflammatory damage to the brain. Therefore, in dealing with CNS-invading pathogens, the host actually needs to reasonably balance the battle between the pathogen and the host, and this effective quenching of the neuroinflammatory responses should be concerned.

As already known, microRNAs (miRNAs) are an important subgroup of non-coding RNAs that negatively modulate messenger RNA (mRNA) translation by binding with the 3’-untranlated regions (3’UTRs) of the respective target mRNAs [11], thus being recognized as important negative-regulatory elements. Increasing studies have proved that aberrant miRNA expression could lead to the subversion of inflammation by regulation of target mRNAs [12], among which some miRNAs act as proinflammatory regulators, such as miR-126-5p positively regulating the inflammatory response by targeting cylindromatosis (CYLD) in monocytes of chronic HIV-1 patients [13], miR-301a in intestinal epithelial cells reduces the anti-proliferation factor 1 (BTG1), thus promoting the inflammation and tumorigenesis in IBD patients [14]. In contrast, some miRNAs participate in feedback attenuation of the ongoing inflammatory response; for example, miR-31 reduces inflammation in sepsis by targeting HMOX1, and miR-718 inhibits proinflammatory cytokines via targeting phosphatase and tensin in LPS-induced inflammation [15,16]. Likewise, in facing bacterial infection, miR-302b provides negative feedback to Toll-like receptors (TLR)-mediated inflammation in *Pseudomonas aeruginosa* infection [17], and downregulation of miR-let-7f leads to the attenuation of inflammation after *Mycobacterium tuberculosis* (*Mtb*) infection [18]. Since our previous whole transcriptome sequencing also revealed a large number of differential miRNAs in BMECs in response to meningitic *E. coli* challenge, we therefore sought to explore how these host miRNAs work to modulate or balance the infection-induced inflammatory response at the entry of the BBB. 

In the present study, we reported the quenching of bacterial-induced neuroinflammation at the BBB in response to meningitic *E. coli* infection via miRNA-mediated signaling. We demonstrated in vivo and in vitro that meningitic *E. coli* infection of BMECs could downregulate miR-19b-3p that led to attenuated production of proinflammatory cytokines and chemokines via the TNFAIP3/NF-κB axis. Moreover, in vivo injection of miR-19b-3p mimics prior to meningitic *E. coli* challenge further augmented inflammation and deteriorated overall health condition of the mice. These findings together reveal that miR-19b-3p itself could act to amplify the neuroinflammatory responses at the BBB, and when suffering from meningitic *E. coli* infection, the host could in turn initiate an effective “quenching mechanism” by attenuating miR-19b-3p/TNFAIP3/NF-κB signaling, which results in alleviation of the proinflammatory responses induced by bacteria at the entry of the BBB. 

## 2. Results

### 2.1. Downregulation of miR-19b-3p in Human BMECs (hBMECs) upon Meningitic E. coli PCN033 Infection

**Figure 1:**
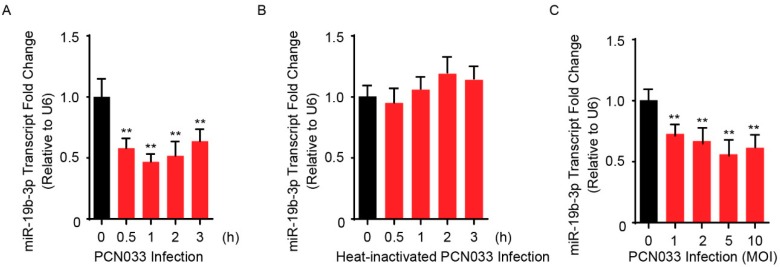
Meningitic *Escherichia coli* PCN033 infection decreases the endogenous miR-19b-3p levels in human brain microvascular endothelial cells (hBMECs). (**A**) The significant decrease of miR-19b-3p transcription in hBMECs in response to PCN033 infection (Multiplicity of Infection, MOI = 10) at different time points. (**B**) Heat-inactivated PCN033 at a MOI of 10 was unable to decrease miR-19-3p levels in hBMECs at indicated time points. (**C**) The significant reduction of miR-19b-3p levels in hBMECs upon PCN033 infection at indicated MOI for 3 h. Data represent mean ± standard deviation (SD) from three independent experiments. U6 was used as the reference control for the miR-19b-3p quantitation. ** p < 0.01.

Our previous RNAs transcriptional profilings of meningitic *E. coli*-infected hBMECs revealed that numbers of miRNAs were differentially expressed in hBMECs in response to the infection, and one of these, miR-19b-3p, showed a significant reduction upon infection based on sequencing [19]. Here, we further verified this expression alteration of miR-19b-3p in hBMECs by the stimulation of meningitic *E. coli* PCN033 for different time points, and quantitative real-time PCR results showed that PCN033 induced a significant decrease of miR-19b-3p expression along with the infection (Figure 1A), and this decrease of miR-19b-3p was not observed in hBMECs in response to heat-inactivated PCN033 (Figure 1B). We moreover observed the significant decline of miR-19b-3p level in response to different dosages of PCN033 challenge (Figure 1C). These results together, in line with our RNA-sequencing data, evidenced that meningitic *E. coli* infection can downregulate miR-19b-3p expression in hBMECs. Since miR-19b-3p has been proved to be a regulator of inflammatory response [20], and that we also previously demonstrated the PCN033-induced neuroinflammation, we therefore hypothesized that miR-19b-3p might be functionally involved in meningitic *E. coli*-induced neuroinflammatory responses, which should be further addressed. 

### 2.2. MiR-19b-3p Can Facilitate PCN033-Triggered Inflammatory Response

**Figure 2:**
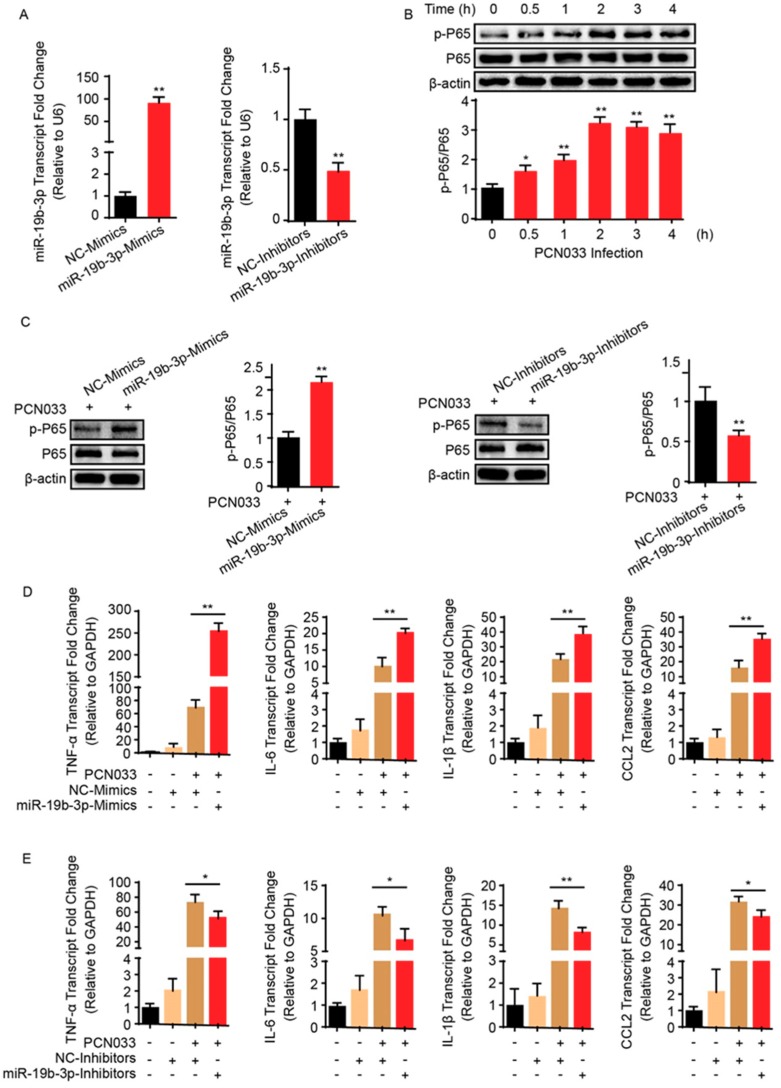
MiR-19b-3p enhances meningitic *E. coli* PCN033-induced inflammatory responses. (**A**) The significant increase or decrease of endogenous miR-19b-3p level in hBMECs by transfection of the synthetic miR-19b-3p mimics and inhibitors (both at final concentration of 50 nM), respectively. (**B**) The significant time-dependent activation of NF-κB p65 subunit in hBMECs in response to PCN033 challenge, determined by Western blot. The β-actin protein served as the loading control, and densitometry was performed to analyze the blotting bands. (**C**) Effects of miR-19b-3p mimic or inhibitor transfection (at 50 nM) on the phosphorylation of NF-κB p65 subunit in hBMECs in response to PCN033 infection (at Multiplicity of Infection (MOI) of 10 for 3 h). The β-actin was used as loading control, and densitometry was performed to analyze the difference among treatments. (**D**,**E**) Effects of the miR-19b-3p mimic or inhibitor transfection on the PCN033-induced production of cytokines and chemokines determined by qPCR. Data represent mean ± SD from three independent experiments. GAPDH was used as the internal reference. * p < 0.05; ** p < 0.01.

Bacterial meningitis is usually accompanied by the BBB permeability alteration, as well as the endothelial inflammatory activation that is supported by release of proinflammatory cytokines and chemokines upon infection [21]. To investigate the role of miR-19b-3p in meningitic *E. coli* PCN033-mediated neuroinflammation, the miR-19b-3p levels were manipulated by transfection of the synthetic miR-19b-3p mimics and inhibitors, which exhibited significant increase or decrease of endogenous miR-19b-3p level in hBMECs (Figure 2A). Since we observed a significant time-dependent activation of NF-κB p65 subunit in hBMECs along with the infection (Figure 2B), which was exactly consistent with our previous data [21,22], we next investigated the effects of miR-19b-3p overexpression or inhibition on the phosphorylation of NF-κB p65 subunit, as well as on the generation of proinflammatory cytokines and chemokines in response to the infection. We observed that overexpression of miR-19b-3p via mimic transfection for 36 h significantly increased the phosphorylation of p65 induced by PCN033, while vice versa, the inhibition of miR-19b-3p significantly reduced p65 activation, as compared with their respective controls (Figure 2C). Moreover, the expression of selected cytokines and chemokines induced by PCN033 infection, i.e., TNF-α, IL-6, IL-1β, and CCL2, were further significantly increased upon miR-19b-3p mimic transfection, as compared to the infection with control mimics (Figure 2D), while their expression were significantly decreased when treated with miR-19b-3p inhibitors (Figure 2E). These data together indicate a positive regulation of miR-19b-3p to meningitic *E. coli*-induced neuroinflammation.

### 2.3. TNFAIP3 Is a Direct Target of miR-19b-3p in Meningitic E. coli Infection of hBMECs

**Figure 3:**
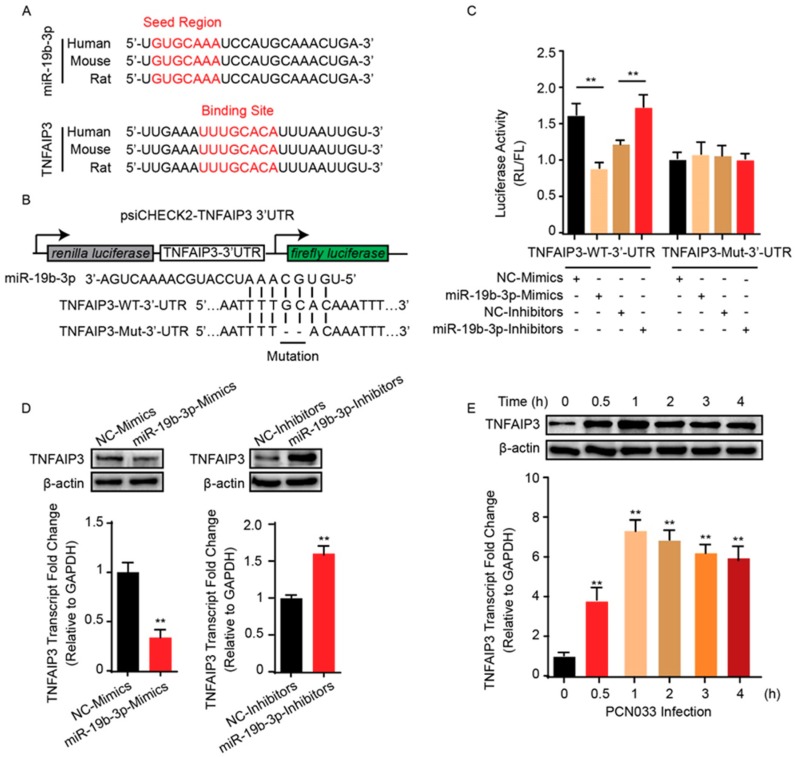
miR-19b-3p negatively regulates the expression of TNFAIP3. (**A**) Conservation of the miR-19b-3p sequence among different species (upper panel), and conservation of the miR-19b-3p target sequence in TNFAIP3 among different species (lower panel). Human, *Homo sapiens*; Mouse, *Mus musculus*; Rat, *Rattus norvegicus*. (**B**) The miRNA response elements (MREs) of miR-19b-3p were shown on the sequence of TNFAIP3 3’-UTR, and mutations were introduced on these MREs. Both wild-type and the mutated sequences were cloned into psiCHECK-2 plasmid. (**C**) 293T cells were co-transfected with miR-19b-3p mimics, miR-19b-3p inhibitors, or their corresponding control oligonucleotide (final concentration at 50 nM), together with the wild-type or mutated TNFAIP3 3’-UTR luciferase reporter plasmid, and renilla luciferase activity was measured and normalized to firefly luciferase activity after 24 h. (**D**) TNFAIP3 protein, as well as mRNA levels, were determined in hBMECs after 24 h of transfection with miR-19b-3p mimics, miR-19b-3p inhibitors, or their corresponding control oligonucleotide (final concentration at 50 nM). (**E**) Western blot and qPCR analyses of TNFAIP3 in hBMECs in response to PCN033 infection. Data represent mean ± SD from three independent experiments. Protein level was normalized to β-actin, and mRNA level as normalized to GAPDH. ** p < 0.01.

An earlier study pointed to TNFAIP3 as one of the potential targets of miR-19b-3p [23]. Since miR-19b-3p was shown to be involved in meningitic *E. coli* infection, whether it also functioned through TNFAIP3 was unclear and needed to be further revealed. Herein, we preliminarily analyzed the binding possibility of miR-19b-3p in TNFAIP3 3’UTR region by using online miRNA target prediction software, such as TargetScan, miRDB, and miRanda. The potential miRNA binding region was found exactly at 3’UTR of TNFAIP3, and this binding site was perfectly aligned with that among different species, including human, mouse, and rat (Figure 3A). To testify the possible regulation between miR-19b-3p and TNFAIP3 in the perspective of meningitic *E. coli* infection, the dual-luciferase reporter plasmid was constructed by cloning TNFAIP3 3’UTR binding region into psiCHECK-2 plasmid (Figure 3B). Likewise, a mutant 3’UTR region was generated and inserted into psiCHECK-2 (Figure 3B). As per the dual-luciferase reporter system shown in Figure 3C, compared with their respective controls, co-transfection of the wild-type 3’UTR reporter plasmid with miR-19b-3p mimics led to a significant decrease of the luciferase activity, while co-transfection with miR-19b-3p inhibitor significantly increased the luciferase activity. These regulatory effects were not observed at all with the TNFAIP3 mutant 3’UTR (Figure 3C). To further authenticate the negative correlation between miR-19b-3p and TNFAIP3, hBMECs were transfected with miR-19b-3p mimics or inhibitors, along with their respective controls, and the endogenous level of TNFAIP3 was determined by quantitative real-time PCR, as well as immunoblotting. In line with luciferase activity data, the passive transfection of miR-19b-3p mimics significantly inhibited TNFAIP3 mRNA and protein expression, while the miR-19b-3p inhibitor transfection led to significantly increased TNFAIP3 expression in both mRNA and protein levels (Figure 3D), further strengthening the negative regulation between miR-19b-3p and its target TNFAIP3. Since meningitic *E. coli* PCN033 induced a significant reduction of miR-19b-3p in hBMECs, we therefore investigated the possible expression change of TNFAIP3 during the infection. As expected, both real-time PCR and Western blotting showed that TNFAIP3 expression exhibited a significant increase in hBMECs along with meningitic *E. coli* infection, which exhibited the exact opposite trend as that of the miR-19b-3p (Figure 3E). Taken together, these data robustly support our narrative that TNFAIP3 is directly and negatively regulated by miR-19b-3p in hBMECs in response to meningitic *E. coli* infection.

### 2.4. TNFAIP3 Negatively Participates the Inflammatory Responses in hBMECs

**Figure 4:**
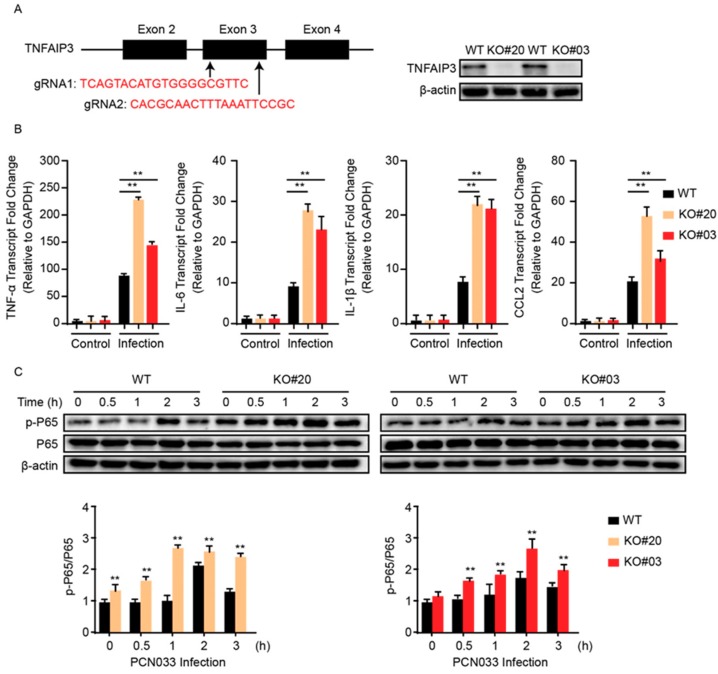
Knock-out of TNFAIP3 significantly increases meningitic *E. coli*-caused neuroinflammatory responses in hBMECs. (**A**) Schematics briefly showing the design of two gRNAs (gRNA1 and gRNA2) in the TNFAIP3 Exon 3 and the identification of TNFAIP3 knock-out (KO) cell lines KO#20 and KO#03 through Western blot analysis. (**B**) qPCR analysis of TNF-α, IL-6, IL-1β, and CCL2 expression in wild-type, TNFAIP KO#20, and TNFAIP KO#03 hBMECs in response to PCN033 infection. Data represent mean ± SD from three independent experiments. GAPDH was used as the internal reference. (**C**) Western blot analysis of the p65 phosphorylation in wild-type, TNFAIP KO#20, and TNFAIP KO#03 hBMECs in response to PCN033 infection. β-actin was used as the loading control, and densitometry was performed to analyze the differences. ** p < 0.01.

To further corroborate that miR-19b-3p is involved in NF-κB signaling via TNFAIP3, we next sought to delete TNFAIP3 in hBMECs through the CRISPR/Cas9 editing approach by using two guide RNAs that target Exon 3 of TNFAIP3 (Figure 4A). Here, several TNFAIP3 knock-out (KO) cell clones, i.e., KO#20 and KO#03, were generated and verified by both sequencing (data not shown) and Western blotting (Figure 4A). We next compared the inflammatory responses of both wild-type and TNFAIP3-KO hBMECs in response to meningitic *E. coli* PCN033 challenge. As shown, both TNFAIP3-KO cell clones (KO#20 and KO#03) showed significant higher levels of proinflammatory factors like TNF-α, IL-6, IL-1β, and CCL2 in response to the infection, as compared to that of the wild-type cells (Figure 4B). We additionally determined the effect of TNFAIP3-KO on the bacterial-induced activation of NF-κB signaling, and the immunoblotting showed that knock-out of TNFAIP3 in hBMECs significantly augmented the p65 phosphorylation as compared to wild-type hBMECs (Figure 4C), suggesting an intensified inflammatory response in TNFAIP3-KO cells along with the infection.

### 2.5. MiR-19b-3p Aggravates PCN033-Induced Inflammatory Responses in Mice

**Figure 5:**
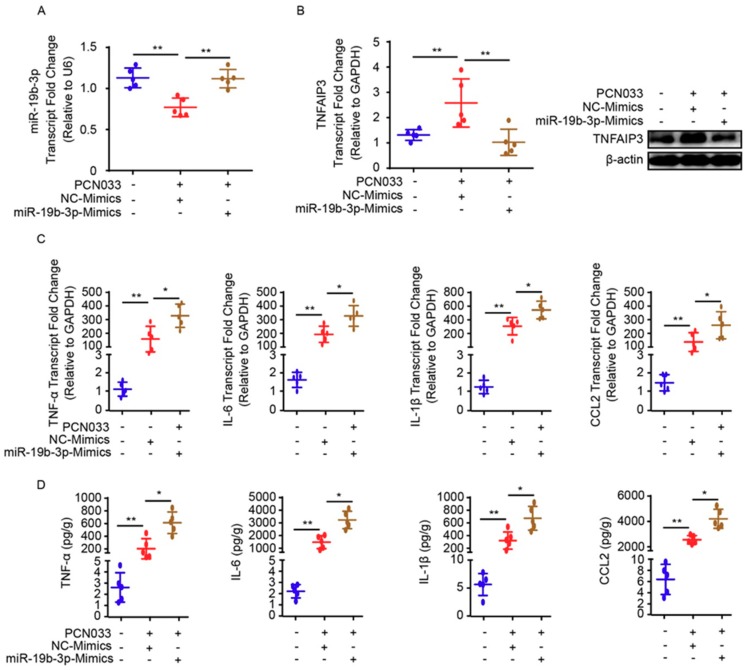
miR-19b-3p mimic pretreatment contributes to neuroinflammatory responses in meningitic *E. coli*-challenged mice. (**A**) qPCR analysis of miR-19b-3p in brain lysates from control mice, PCN033-challenged mice with or without miR-19b-3p mimic pretreatment. U6 was used as the internal reference. Data are presented as mean ± SD from five individual mice. (**B**) The mRNA level of TNFAIP3 in mouse brains from control mice, PCN033-challenged mice with or without miR-19b-3p mimic pretreatment was analyzed by qPCR. GAPDH was used as the internal reference. Data were presented as mean ± SD from five individual mice. The protein level of TNFAIP3 in mouse brains from control mice, PCN033-challenged mice with or without miR-19b-3p mimic pretreatment was additionally analyzed by Western blot. β-actin was used as the loading control. (**C**) qPCR analysis of TNF-α, IL-1β, IL-6, and CCL2 transcription from control mice, PCN033-challenged mice with or without miR-19b-3p mimic pretreatment. GAPDH was used as the internal reference. Data are presented as mean ± SD from five individual mice. (**D**) ECL analysis of TNF-α, IL-1β, IL-6, and CCL2 levels in brain lysates from control mice, PCN033-challenged mice with or without miR-19b-3p mimic pretreatment. Data are expressed as mean ± SD (**n** = 5). * *p* < 0.05; ** *p* < 0.01.

**Figure 6:**
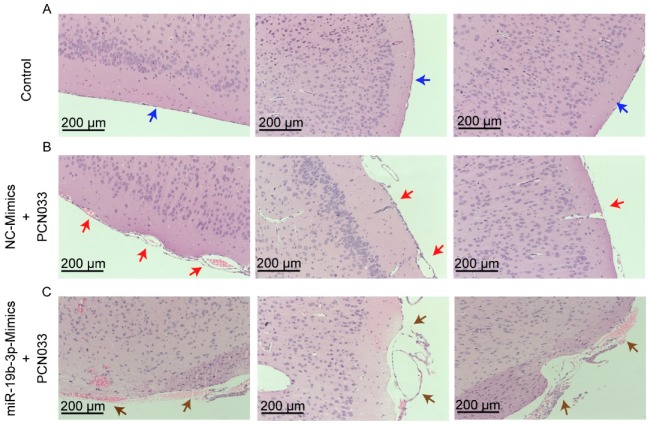
Histopathological analysis of meninges in PCN033-challenged mice with or without miR-19b-3p mimic pretreatment. (**A**) The normal meninges from uninfected mice (blue arrows). (**B**) The meninges from PCN033-challenged mice receiving the control mimics. Red arrows indicate meninges thickening, hyperemia, and inflammatory cell accumulation. (**C**) The meninges from PCN033-challenged mice receiving miR-19b-3p mimic pretreatment, and brown arrows show more severe thickening, hyperemia, and accumulation of inflammatory cells, as compared to the challenged brains with control mimic treatment. Scale bar = 200 μm.

To describe the significance of miR-19b-3p in PCN033-caused neuroinflammation in vivo, the mice model that received meningitic *E. coli* injection was applied. The chemically-modified antisense oligonucleotide miR-19b-3p mimic, which is able to cross the BBB, as demonstrated in previous studies [18,24], was administered intravenously into mice 24 h prior to PCN033 infection to enhance the endogenous expression of miR-19b-3p. The miR-19b-3p mimic injection was well tolerated by mice, without displaying any sign of uneasiness. As shown, at 6 h post-challenge, when mice began to exhibit typical neurological symptoms like overactive, circling, trembling, paddling, and opisthotonos [21], a significant reduction of miR-19b-3p was observed in challenged mice brains, while the miR-19b-3p mimic pretreatment could obviously restore the endogenous miR-19b-3p levels in the challenged mice (Figure 5A). Correspondingly, the TNFAIP3 mRNA transcription, as well as protein expression level in the brain, were significantly increased upon PCN033 infection, while the miR-19b-3p pretreatment significantly recovered this TNFAIP3 upregulation (Figure 5B). As significant upsurge of inflammatory cytokines is evident for neuropathological and cytotoxic conditions during PCN033 infection [21], we subsequently tested the expression of those proinflammation cytokines and chemokines, including TNF-α, IL-6, IL-1β, and CCL2, in PCN033-challenged mice with or without mimic treatment, and found that the miR-19b-3p mimic pretreatment significantly aggravated the infection-induced inflammatory responses (Figure 5C). Moreover, by using ECL Multi-Array® assay, we evaluated in vivo the effect of miR-19b-3p mimic injection on the production of proinflammatory cytokines and chemokines, i.e., TNF-α, IL-6, IL-1β, and CCL2, in the perspective of PCN033-challenged mice. As shown, we observed the significant upregulation of these cytokines and chemokines in PCN033-infected mice brains, which is consistent as our previous study [21], and notably, a further high levels of cytokines and chemokines were observed in the challenged mice group with miR-19b-3p mimic pretreatment, compared with the challenged mice receiving control mimics (Figure 5D). Besides, histopathological examination was applied and showed the obvious congestion and thickening of the meninges in mice after PCN033 infection, and this histologic lesion became exacerbated during the infection when mice received the miR-19b-3p mimic, demonstrated by a further thickening, congestion, as well as meninges damage (Figure 6A–C). These in vivo results, in line with the in vitro data, reveal that miR-19b-3p could exert as an amplifier of the bacterial-induced inflammation via decreasing the negative inflammatory regulator TNFAIP3—and upon meningitic *E. coli* challenge, the brain endothelium could initiate the quenching mechanism by attenuating the miR-19b-3p-midiated proinflammatory signaling.

## 3. Discussion

BMECs are recognized as key components of the host’s BBB, which is characterized by the presence of several tight junction proteins, as well as low pinocytosis, constituting the key restricting barrier preventing circulating pathogens from entering the brain [25]. In meningitis pathophysiology, BBB disruption is an obligatory outcome, accompanied by acute inflammatory responses, with the release of multiple inflammatory factors such as TNF-α, IL-1β, IL-6, IL-17A, MCP1, and GRO-α [21]. Previously, several in vitro and in vivo studies discussed the potential role of BMECs in direct limitation of BBB permeability [21,26]; however, it is unclear as to what the quenching mechanisms of BMECs are in limiting acute inflammatory responses.

In early studies, we reported that PCN033 was a meningitis-associated *E. coli* strain isolated from diseased pig brain tissues, and consequently verified the establishment of PCN033 invasion of brain tissues by compromising the BBB permeability [21,22]. Thereafter, a step further to highlight the host narrative against PCN033 infection, a comprehensive RNA-sequencing analysis was carried out [19], which revealed a notable differential expression of multiple miRNA profiles. Out of these miRNA profiles, we selected miR-19b-3p for further experimentation due to its subsequent lower expression according to our RNA-sequencing data. The miR-19b-3p is a member of miR-17/92 clustered with miR-17, miR-18a, miR-19a, miR-20a, miR-19b-1, and miR-92-1, firstly originated from human B-cell lymphoma samples [27], which has decisive biological significance in the development of cancer and many other pathological pathways [28]. Some recent reports designated that miR-19b-3p acted as a promising diagnostic tool in multiple disease conditions [29,30] and as a modulator of innate immune response as well [24]. However, the inflammation-related function of miR-19b-3p, particularly the in context of bacterial infection, as well as CNS disorder, has rarely been studied in the past. In the current study, we revealed a quenching working mechanism involving miR-19b-3p during the meningitic *E. coli*-mediated neuroinflammatory process. We found that hBMECs could downregulate miR-19b-3p expression, which led to attenuated generation of proinflammatory cytokines and chemokines in response to meningitic *E. coli* infection by upregulating the expression of TNFAIP3, a direct target of miR-19b-3p that exerts the negative regulation of the neuroinflammatory responses. To our knowledge, this is probably the first direct study reporting a miRNA in BMECs participating as the host anti-inflammatory mechanism in response to CNS infection, which shall further strengthen the current knowledge that BMECs, other than astrocytes and pericytes, also exert significant roles in the modulation of inflammatory response at the pathogen entry of the BBB.

TNFAIP3 has been reported to be a ubiquitin-dependent anti-inflammatory signaling molecule that is involved in several intracellular signaling pathways [31,32]. It is expressed in different cell lines, serving as a key element in the negative feedback regulation of the NF-κB signaling pathways [33]. TNFAIP3 is generally characterized by two functional domains—one N-terminal domain with deubiquitinating activity, and one C-terminal zinc finger domain [31]. In the NF-κB signaling pathway, TNFAIP3 resists TNF-induced NF-κB signaling by replacing K48-linked polyubiquitin chains with K63-linked polyubiquitin chains to terminate the receptor interacting protein1 (RIP1) interaction with NF-κB essential modulator (NEMO) [34], thus ultimately limiting the inflammatory responses induced by many pattern recognition receptors such as TLRs [32,35]. The preventive roles of TNFAIP3 have been widely reported in a number of systemic inflammatory diseases, such as rheumatoid arthritis, autoimmune diseases, gastrointestinal and hepatic disorders, psoriasis, aging, and cancer [36], and also in different viral infections such as measles virus [37], influenza virus [38], and parasitic infection like *Leishmania donovani* [38]. In the interface of host–microbe interaction, TNFAIP3 most likely acts as an inhibitory modulator in disease pathophysiology [39]. Besides, recent in vivo studies also revealed that TNFAIP3 was functionally important in different cells, such as B cells, dendritic cells, keratinocyte, etc., for anti-inflammatory and autoimmune pathophysiology [33], and TNFAIP3 knock-out mice died in the 3–4 weeks after birth due to impulsive inflammatory responses [40]. Despite these findings, the CNS-related TNFAIP3 functionalities in the perspective of bacterial infections have rarely been acknowledged in the past. In our work, we observed the upregulation of TNFAIP3 in hBMECs, mediated by meningitic *E. coli* infection-caused decrease of miR-19b-3p. We proposed that an enhanced TNFAIP3 expression was an essential brake to restrict the bacterial-induced neuroinflammation, which is a step forward to curb aberrant inflammatory responses. We subsequently knocked out TNFAIP3 from hBMECs by applying the CRISPR/Cas9 approach and demonstrated a significant increase of proinflammatory cytokine and chemokines, which is similar to those with miR-19b-3p mimic transfection during the infection. Noticeably, a similar TNFAIP3-associated anti-inflammatory mechanism triggered by miR-let-7 has also been reported in the infection of *Mycobacterium tuberculosis* [18], further emphasizing the concept that TNFAIP3 could act as the key modulator between the pathogen and the host that operate the infection-triggered inflammation.

In conclusion, we provided in vivo and in vitro pieces of evidence that decreased miR-19b-3p mediated the attenuation of meningitic *E. coli*-induced neuroinflammation by targeting TNFAIP3, a potential negative regulator of the NF-κB signaling pathway. This decrease of miR-19b-3p-mediated inflammatory responses against bacteria is an effective quenching mechanism that is initiated by the host to protect the CNS from inflammatory damages, and more importantly, the miR-19b-3p may be considered a potential therapeutic target towards meningitic bacterial-induced neuroinflammation.

## 4. Materials and Methods 

### 4.1. Bacterial Strains, Cell Culture, and Infection

PCN033, a previously preserved cerebrospinal fluid *E. coli* isolate in our lab, was characterized as a highly virulent extraintestinal pathogenic *E. coli* strain, and has also been previously evidenced as a virulent meningitis-causing *E. coli* [10,19,41]. PCN033 strain was routinely grown in Luria–Bertani (LB) medium in aerobic conditions at 37 °C. 

The human BMECs (defined as hBMECs) were kindly provide by Prof. Kwang Sik Kim from Johns Hopkins University School of Medicine [26,42], and cultured routinely in RPMI1640 medium supplemented with 10% fetal bovine serum, 1 mM sodium pyruvate, 2 mM L-glutamine, nonessential amino acids, essential amino acids, penicillin and streptomycin (100 U/mL), and vitamins in a 37 °C incubator under 5% CO_2_. In some experiments, the confluent hBMEC monolayer was gently washed in Hank’s Balanced Salt Solution (Corning Cellgro, Manassas, VA, USA) and starved in serum-free medium (1:1 mixture of Ham’s F-12 and M-199) for 12–16 h before bacterial infection. 

For bacterial challenge, the cells were infected with PCN033 at a MOI of 10 (approximately 10^8^ colony-forming units (CFUs) per 100 mm dish). In some assays, the cells were transfected with specific mimics or inhibitors prior to bacterial challenge.

### 4.2. Reagents and Antibodies

The anti-TNFAIP3 antibody was purchased from Arigo Biolaboratories (Hamburg, Germany). Anti-β-actin antibody was from HuaAn Biotechnology Co., Ltd. (Hangzhou, China). Anti-NF-κB p65, anti-phospho-p65, HRP-conjugated anti-rabbit IgG, and anti-mouse IgG were obtained from Cell Signaling Technology (Danvers, MA, USA). Transfection reagent Lipofectamine^TM^ 3000 was purchased from Invitrogen (Carlsbad, CA, USA). The all-in-one CRISPR/Cas9 plasmid pYSY-spCas9-sgRNA-Puro for gene editing was purchased from YSY Biotech Co. LTD. (Nanjing, China). RNA oligonucleotides, including miR-19b-3p mimics (double-stranded RNA oligonucleotides), inhibitors (single-stranded chemically modified oligonucleotides), and their respective control oligonucleotides, were commercially available at GenePharma (Shanghai, China). Sequences of all oligonucleotides are listed in Table 1.

### 4.3. Plasmid Construction

The 3’-UTR of TNFAIP3 was PCR amplified from hBMEC cDNA with specific primers (Forward: 5’-CCGCTCGAGGTCTGTACTTATGGCCTGAAAATAT-3’ Reverse: 5’-AAAGCGGCCGCCTTGAAGAAATCCAACAAAGAATAG-3’), and then cloned into the 3’ terminal of Renilla luciferase in dual-luciferase reporter plasmid psiCheck-2 (Promega Madison, WI, USA) at the *Xho*I and *Not*I sites. The TNFAIP3 3’-UTR mutant construct was similarly generated with specific point mutation by using overlapping extension PCR with primers as follows: Forward: 5’-AAATTTTACAAATTTAATTGTCCCTAATAGAAAG-3’, and Reverse: 5’-TTAAATTTGTAAAATTTCAAATACTTTTTATAATAA-3’. All constructs developed were confirmed by sequencing.

### 4.4. Transfection

The hBMECs were seeded in 6-well plates and cultured up to 80% of confluence, and then transfected with siRNA or overexpression plasmid by using Lipofactamin^TM^ 3000, according to the manufacturer’s instructions. Briefly, 2 μg of plasmid, 10 μL of P3000, 7.5 μL of Lipo3000, and 500 μL of Opti-MEM were mixed gently and then incubated at room temperature for 15 min. The mixture was then added dropwise into cells, followed by 24–48 h of incubation at 37 °C with 5% CO_2_. To maintain the transfection-positive cells, the culture medium was removed and replaced by complete medium containing puromycin (100 μg/mL). In some assays, the medium was replaced by the serum-free medium after 36 h post-transfection, and cells were subsequently infected with bacteria at MOI of 10 for the indicated times.

### 4.5. TNFAIP3 Knocking out via CRISPR/Cas9 Genomic Editing

The human TNFAIP3 sgRNA1 (5’-TCAGTACATGTGGGGCGTTC-3’) and sgRNA2 (5’-CACGCAACTTTAAATTCCGC-3’) were synthesized and inserted into the all-in-one CRISPR/Cas9 vector to generate the pYSY-spCas9-TNFAIP3-sgRNA-Puro plasmid. Cells were seeded in 6-well plates at a density of 2 × 10^5^ cells per well for 24 h and then subjected to transient transfection with 2 μg plasmid. After 24 h of incubation, fresh medium containing 200 ng/mL puromycin was added and incubated for another 48 h. The surviving cells were then transferred into 96-well plates with limiting dilution method until the single-cell clone formation. Genomic DNA from single-cell clone was extracted and PCR was performed to amplify the target region with the following primers: 5’-TGAAATATCAGTTTGCCCTTG-3’ (forward) and 5’-GGCTAAGCGAAGCATACTCAA-3’ (reverse). The positive knock-out cells were identified by sequencing. 

### 4.6. Dual-Luciferase Reporter Assay

HEK293T cells were co-transfected with 200 ng of the constructed luciferase reporter plasmid, along with the miR-19b-3p mimics or the respective negative control mimics (at final concentration of 50 nM) as per the abovementioned method. The dual-luciferase reporter assay was then applied to measure the Renilla luciferase, as well as Firefly luciferase, activities after 36 h of incubation, following the manufacturer’s instructions (Promega, Madison, WI, USA). Relative luciferase activity was calculated by the ratio of reporter activity (Renilla fluorescence) to that of control activity (Firefly fluorescence), and the results were shown as the representative of three independent assays.

### 4.7. In Vivo Mice Infection Assays

The animal assays performed in this study were based on the guidelines approved by the China Regulations for the Administration of Affairs Concerning Experimental Animals (1988) and Regulations for the Administration of Affairs Concerning Experimental Animals in Hubei (2005) (Project No. 00279539 and Animal Welfare Assurance No. 190814). All of the procedure and handling techniques were as per suggested by the Committee for Protection, Supervision and Control of Experiments on Animals guidelines of Huazhong Agricultural University (Permit No. SYXK2018-0070). All efforts were made to treat the experimental animals in this study ethically, and to minimize suffering. The 28-day-old SPF KM mice were obtained from the Center for Disease Control in Hubei province of China and were randomly assigned to three groups, including control group (group 1) which received the phosphate-buffered saline (PBS) treatment, negative mimics group (group 2, named NC-PCN033) which received negative control mimic treatment and meningitic *E. coli* PCN033 challenge as well, and the experimental group (group 3, named Mimics-PCN033) which received both miR-19b-3p mimics and PCN033 challenge. For the mimics, 10 ODs of the mimic (600 μL/mice) were injected respectively through tail vein 24 h prior to bacterial challenge [24]. Mice were intravenously inoculated with 1 × 10^7^ CFUs of PCN033 strain or equal volume of PBS. After 6 h post-infection, when CNS disorder symptoms appeared, challenged mice were sacrificed and brain samples were collected for further assays. 

### 4.8. RNA Extraction and Quantitative Real-Time PCR

Total RNAs from hBMECs were extracted by using the TRIzol reagent (Invitrogen), and aliquots (1 μg) of the total RNAs in each sample were subjected to cDNA synthesis with the PrimeScript^TM^ RT kit (Takara Bio Inc., Japan) for miRNAs and the Vanzyme® kit for mRNAs, respectively, following the manufacturers’ instructions. Quantitative real-time PCR was carried out with a qTOWER3/G quantitative real-time PCR thermal cycler (Analytik jena, Jena, Germany) by using Power SYBR Green PCR master mix (Applied Biosystems, Foster City, CA, USA), according to the instructions. The amplification was implemented at 50 °C for 2 min, 95 °C for 10 min, followed by 40 cycles of 95 °C for 15 s and 60 °C for 1 min. To ensure primer specificity at the target genes, the products were subsequently applied to a melt curve stage with denaturation at 95 °C for 15 s, annealing at 60 °C for 1 min, and slow dissociation by increasing from 60 to 95 °C at 0.05 °C/s. All mRNA target expressions were normalized to GAPDH, and the miRNA expression was normalized to U6. The quantitative PCR assays were performed in triplicate. All primers used here are listed in Table 1.

### 4.9. Immunoblotting

The brain tissues or the infected/transfected hBMECs were homogenized or lysed in RIPA buffer with protease inhibitor cocktail (Sigma-Aldrich, USA), sonicated and centrifuged at 12,000 rpm at 4 °C for 10 min to remove insoluble debris. The supernatant was then separated for immunoblotting analyses as previously described [21,43]. The bicinchoninic acid (BCA) protein assay kit (Thermo Scientific) was used to measure protein concentration in each sample. The blots were densitometrically analyzed by using ImageLab software version 5.2.1 (Bio-Rad, Hercules, CA, USA), and the results were exhibited as relative immunoreactivity of each protein normalized to the respective loading control.

### 4.10. Electrochemiluminescence (ECL) Assay

Challenged mice brain tissues from each group were collected and lysed in RIPA buffer supplemented with protease inhibitor. The brain lysates were centrifuged at 12,000 rpm for 10 min to eliminate debris, and the supernatant was applied to ECL assays to measure the levels of proinflammatory cytokines and chemokines, including IL-1β, IL-6, TNF-α, and CCL-2, by using MSD V-Plex™ Proinflammatory Panel 1 (Mouse) Calibrator Blend ELISA (Meso Scale Discovery, Meso Scale Diagnostics, Rockville, MD, USA), according to the guidelines provided. 

### 4.11. Histopathological Examination

Mice brains of all groups were collected and fixed in 4% formaldehyde solution for over 48 h. The paraffin-embedded sections up to 6 μm were subsequently prepared and subjected to the standard protocol of hematoxylin and eosin (H&E) histopathological staining [44].

### 4.12. Statistical Analysis

All data above are expressed as mean ± standard deviation (mean ± SD) unless otherwise specified. The variation between two groups was analyzed by Student’s *t*-test, and difference significance was compared by one-way or two-way analysis of variance (ANOVA) embedded in GraphPad Prism Ver. 6.02. For all tests, *p* < 0.05 (*) was considered significant, while *p* < 0.01 (**) was assigned to extremely significant differences.

## Figures and Tables

**Table 1:** Primers for qPCR.

Primer Name	Sequence (5’ to 3’)
Human TNFAIP3 (Forward)	TCCTCAGGCTTTGTATTTGAGC
Human TNFAIP3 (reverse)	TGTGTATCGGTGCATGGTTTTA
Mouse TNFAIP3 (Forward)	ACAGTGGACCTGGTAAGAAAACA
Mouse TNFAIP3 (Reverse)	CCTCCGTGACTGATGACAAGAT
Human TNF-α (Forward)	CCTCTCTCTAATCAGCCCTCTG
Human TNF-α (Reverse)	GAGGACCTGGGAGTAGATGAG
Mouse TNF-α (Forward)	CCCTCACACTCAGATCATCTTCT
Mouse TNF-α (Reverse)	GCTACGACGTGGGCTACAG
Human IL-6 (Forward)	ACTCACCTCTTCAGAACGAATTG
Human IL-6 (Reverse)	CCATCTTTGGAAGGTTCAGGTTG
Mouse IL-6 (Forward)	TAGTCCTTCCTACCCCAATTTCC
Mouse IL-6 (Reverse)	TTGGTCCTTAGCCACTCCTTC
Human IL-1β (Forward)	ATGATGGCTTATTACAGTGGCAA
Human IL-1β (Reverse)	GTCGGAGATTCGTAGCTGGA
Mouse IL-1β (Forward)	GCAACTGTTCCTGAACTCAACT
Mouse IL-1β (Reverse)	ATCTTTTGGGGTCCGTCAACT
Human CCL2(Forward)	CAGCCAGATGCAATCAATGCC
Human CCL2 (Reverse)	TGGAATCCTGAACCCACTTCT
Mouse CCL2 (Forward)	TTAAAAACCTGGATCGGAACCAA
Mouse CCL2 (Reverse)	GCATTAGCTTCAGATTTACGGGT
Human GAPDH(Forward)	CAACAGCCTCAAGATCATCAG
Human GAPDH (Reverse)	GAGTCCTTCCACGATACCA
Mouse GAPDH (Forward)	AGGTCGGTGTGAACGGATTTG
Mouse GAPDH (Reverse)	TGTAGACCATGTAGTTGAGGTCA
Human U6 (Forward)	CTCGCTTCGGCAGCACA
Human U6 (Reverse)	AACGCTTCACGAATTTGCGT
Mouse U6 (Forward)	CTCGCTTCGGCAGCACA
Mouse U6 (Reverse)	AACGCTTCACGAATTTGCGT
